# Beta Cell Dysfunction in Youth- and Adult-Onset Type 2 Diabetes: An Extensive Narrative Review with a Special Focus on the Role of Nutrients

**DOI:** 10.3390/nu15092217

**Published:** 2023-05-07

**Authors:** Anastasios Serbis, Vasileios Giapros, Konstantinos Tsamis, Foteini Balomenou, Assimina Galli-Tsinopoulou, Ekaterini Siomou

**Affiliations:** 1 Department of Pediatrics, School of Medicine, University of Ioannina, St. Niarhcos Avenue, 45500 Ioannina, Greece; eksiomou@uoi.gr; 2 Neonatal Intensive Care Unit, School of Medicine, University of Ioannina, St. Νiarhcos Avenue, 45500 Ioannina, Greecefaybal18@yahoo.com (F.B.); 3 Department of Physiology, Faculty of Medicine, School of Health Sciences, University of Ioannina, St. Niarhcos Avenue, 45500 Ioannina, Greece; 4 Second Department of Pediatrics, School of Medicine, Faculty of Health Sciences, Aristotle University of Thessaloniki, AHEPA University Hospital, Stilponos Kyriakidi 1, 54636 Thessaloniki, Greece; agalli@auth.gr

**Keywords:** type 2 diabetes, youth, adolescents, young adults, β-cell failure, insulin

## Abstract

Traditionally a disease of adults, type 2 diabetes (T2D) has been increasingly diagnosed in youth, particularly among adolescents and young adults of minority ethnic groups. Especially, during the recent COVID-19 pandemic, obesity and prediabetes have surged not only in minority ethnic groups but also in the general population, further raising T2D risk. Regarding its pathogenesis, a gradually increasing insulin resistance due to central adiposity combined with a progressively defective β-cell function are the main culprits. Especially in youth-onset T2D, a rapid β-cell activity decline has been observed, leading to higher treatment failure rates, and early complications. In addition, it is well established that both the quantity and quality of food ingested by individuals play a key role in T2D pathogenesis. A chronic imbalance between caloric intake and expenditure together with impaired micronutrient intake can lead to obesity and insulin resistance on one hand, and β-cell failure and defective insulin production on the other. This review summarizes our evolving understanding of the pathophysiological mechanisms involved in defective insulin secretion by the pancreatic islets in youth- and adult-onset T2D and, further, of the role various micronutrients play in these pathomechanisms. This knowledge is essential if we are to curtail the serious long-term complications of T2D both in pediatric and adult populations.

## 1. Introduction

Type 2 diabetes mellitus (T2D) is a heterogeneous disorder characterized by hyperglycemia caused by the combination of an increasing insulin resistance together with a gradual β-cell failure [1]. Traditionally a disease of adults, T2D has been increasingly recognized in youth, especially among minority ethnic groups [2,3,4]. Since T2D is mostly obesity-driven, a further rise in T2D incidence and prevalence is expected in the years to come due to the COVID-19 pandemic and the observed rise in both adult [5] and pediatric obesity [6]. Indeed, based on various estimates, a fourfold increase in the prevalence of youth-onset T2D is predicted in the United States (U.S.) and other parts of the world by 2050, especially among racial and ethnic minority youth [7].

The clarification of youth-onset T2D pathogenesis is important since it has been demonstrated that it is a much more worrisome form of diabetes with rapidly progressive β-cell decline, and increased treatment failure rates leading to early complications [8,9,10,11,12]. Both progressive insulin resistance related to increased central adiposity and defective β-cell function are required for the development of T2D [13]. Obviously, nutritional habits both in youth and in adults are important determinants of the individual’s metabolic status and a chronic imbalance between caloric intake and expenditure leads to obesity. Nutrition and nutrients have been studied regarding their effect on adipose tissue accumulation and insulin resistance development [14,15]. In addition, micronutrient intake and the serum or tissue levels of certain elements have been shown to be involved in β-cell function per se and in insulin production derangements [16].

Regarding the pathophysiology of β-cell failure in T2D, several possible mechanisms have been implicated, affecting the secretory rate of the individual β-cells and/or their number and size [17,18,19]. Such defects could be the result of genetic, epigenetic, or environmental factors acting both prenatally and postnatally on the developing pancreatic tissue and affecting β-cell differentiation, multiplication, insulin production, and cell death. Furthermore, glucotoxicity, lipotoxicity, endoplasmic reticulum (ER) stress, mitochondrial dysfunction, inflammation, and other factors exacerbate β-cell dysfunction leading to a vicious circle. Abnormal micronutrient levels have been shown to play an important role in several of these mechanisms. 

The aim of this narrative review was to summarize which fundamental causative factors have been shown to be implicated in defective pancreatic insulin secretion in youth- and adult-onset T2D and, further, to examine which of these factors are influenced by abnormal levels of specific nutrients. This knowledge is essential if we are to curtail the serious long-term complications of T2D both in pediatric and adult populations.

## 2. Materials and Methods

**Figure 1:**
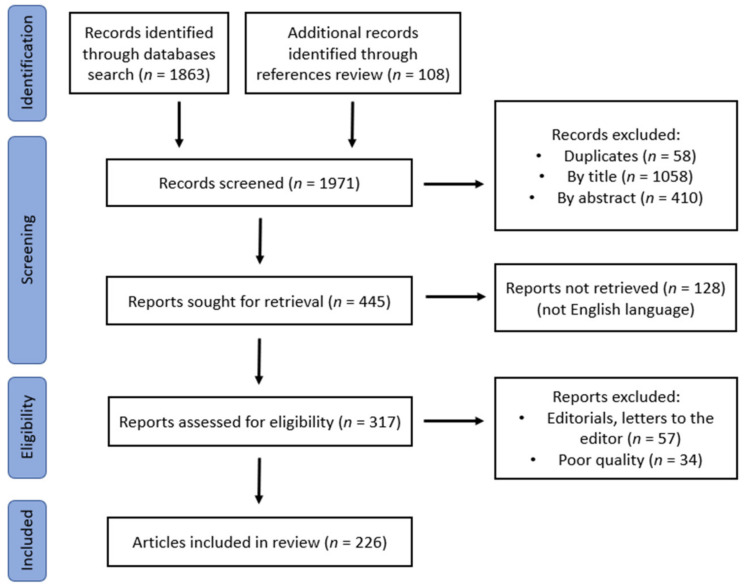
Flowchart of the literature review procedure (record identification, screening, eligibility, and final inclusion).

A literature search on the PubMed/Medline database was conducted, referring to manuscripts/studies between 1980 and 31 December 2022 to identify relevant papers using the following keywords: “type 2 diabetes”, “β-cell failure”, “youth”, “adolescent”, “young adult”, “nutrients”, “minerals”, “vitamins”, and “trace elements”. Exclusion criteria were non-English papers, a publication date before 1980, as well as studies referring exclusively to insulin resistance and obesity or metabolic syndrome. Clinical case reports, case series, observational studies, and systematic reviews were all included in the initial evaluation. Duplicates were identified by title, and relevance was initially examined through title and abstract screening. Full-text articles of all the relevant studies were retrieved and reviewed. A manual search of the references from the retrieved articles led to the identification of further relevant papers that were also included (Figure 1).

## 3. Results

The initial literature search identified 1863 records, of which 58 were excluded as duplicates. In addition, 1058 were deemed irrelevant based on the titles, and a further 401 were rejected based on the abstracts. Furthermore, 128 reports were not available in the English language and were equally excluded. After a manual check of the reference lists of the retrieved reports, 108 additional records were deemed relevant. During the eligibility checking process, 91 reports were also excluded. In the end, 226 articles were considered pertinent and were included in the current review. In the Discussion section, the results are presented categorized according to the possible pathomechanisms involved in β-cell failure related to nutrients, after a brief description of normal β-cell function (Figure 1).

## 4. Discussion

### 4.1. Normal Insulin Production by Pancreatic β-Cells

Depending on the body weight, nutrition, and physical activity of an individual, his/her pancreas releases 20–40 units of insulin per day [20]. Almost half of these units are produced as basal insulin and the rest in response to meals. Basal insulin release in normal subjects shows pulsatility with two major frequencies, one with a period of almost 2 h [21], and the other with a period of 4–6 min [22]. After intravenous glucose administration, β-cells respond by a rapid first phase insulin production that lasts for 5–8 min. A second phase of insulin production follows which is of a lower amplitude but of longer duration [23]. Insulin production after glucose ingestion reaches a peak after almost 60–90 min and returns to basal production after 4–5 h [24]. In addition, more than 50 years ago, Perley et al. showed that the amount of insulin secreted after intravenous glucose administration is only 30–40% of the insulin secreted after oral administration of an equivalent amount of glucose [25]. Gradually, the two gut peptides responsible for this phenomenon, known as the “incretin effect”, were identified, namely the glucagon-like peptide-1 (GLP-1) and the glucose-dependent insulinotropic peptide (GIP) [26]. These two incretins attach to specific receptors on the surface of β-cells to increase intracellular cyclic adenosine monophosphate (cAMP) levels and, eventually, to potentiate insulin secretion [27].

The major physiological stimulus for insulin release is glucose. In addition, amino acids (mainly arginine, and glutamine plus leucine) and fatty acids (especially short chain fatty acids) increase insulin production by directly acting on β-cells [28,29]. In addition to the macronutrient role in β-cell function, recent research has revealed the importance of vitamins, minerals, and trace elements (micronutrients) in insulin production, such as vitamin D, vitamin A, calcium, zinc, magnesium, iron, cobalt, chromium, iodine, and selenium [16]. Beyond nutrients, a multitude of hormones such as glucagon, gastrin, secretin, cholecystokinin, and vasoactive intestinal polypeptide have a positive effect on insulin production, while others, such as somatostatin and ghrelin, have a negative one [30,31]. In addition, neurotransmitters such as acetylcholine and adrenaline can stimulate insulin secretion via β_2_ receptors, while adrenalin and noradrenaline, acting via α_2_ receptors, inhibit insulin secretion [30,31].

**Figure 2:**
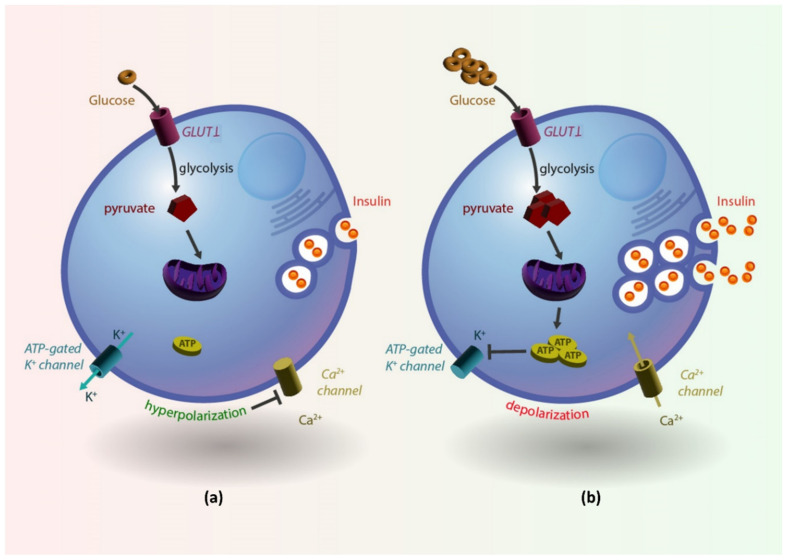
(**a**). Low blood glucose levels lead to low intracellular ATP, open ATP-sensitive K^+^ channels, cell membrane hyperpolarization and, therefore, closed voltage-gated Ca^2+^ channels leading to low intracellular Ca^2+^ concentration and inhibition of insulin secretion. In (**b**), increased blood glucose levels lead to increased ATP production, closure of K^+^ channels, depolarization of the cell membrane with resultant opening of Ca^2+^ channels, influx of Ca^2+^ within the β-cell that leads to insulin granule exocytosis and insulin release into circulation.

At the cellular level, insulin is released by β-cells via a complex process, the so-called “stimulus-secretion coupling”. When blood glucose levels rise, glucose enters the β-cell mainly via the glucose transporter GLUT1 which is localized on the cell surface (Figure 2b). This glucose is rapidly metabolized within the β-cell via oxidative phosphorylation, leading to the formation of pyruvate, which enters the mitochondrion to be oxidized resulting in a net increase in adenosine triphosphate (ATP) concentration. Subsequently, the increased ATP triggers the closure of ATP-sensitive potassium (K^+^) channels and leads to cell depolarization. This β-cell electrical activity facilitates the influx of calcium (Ca^2+^) into the cell by opening the voltage-gated Ca^2+^ channels, which, in turn, triggers the fusion of insulin granules with the plasma membrane in a soluble N-ethylmaleimide-sensitive factor attachment protein receptor (SNARE)-dependent process [32]. Conversely, with low blood glucose levels, insulin secretion by β-cells is minimal since intracellular ATP is low, ATP-sensitive K^+^ channels are open, the cell membrane is hyperpolarized and therefore, voltage-gated Ca^2+^ channels are closed leading to a low intracellular Ca^2+^ concentration and inhibition of insulin secretion (Figure 2a) [32]. 

### 4.2. Clinical Evidence of the β-Cell Dysfunction Role in T2D 

Several epidemiological studies in adult populations have shown that β-cell malfunction is a sine qua non for the clinical appearance of T2D, along with increasing insulin resistance. For example, in an early study including 714 Mexican Americans without diabetes, both decreased insulin secretion and increased insulin resistance were shown to be independently associated with T2D development [33]. Similarly, a study in Pima Indians showed that the gradual progression from NGT to IGT and to overt T2D is characterized by a decrease in insulin secretion and insulin efficacy in glucose disposal [34]. In a more recent prospective study of 6500 British civil servants free of diabetes at the baseline, 505 developed T2D within a median follow-up of 9.7 years. During the five years before the diagnosis, a marked increase in insulin resistance was identified in those that developed T2D with a concomitant initial increase in insulin secretion 3–4 years before the diagnosis, followed by a decrease just before the diabetes diagnosis [35]. This finding suggests an initial β-cell hyperfunction as a compensatory mechanism for the increasing insulin resistance with a subsequent gradual decrease in insulin production, leading to clinically overt diabetes. Similarly, in the Diabetes Prevention Program (DPP) study, a randomized, controlled clinical trial that was conducted in 27 U.S. clinical centers, it was shown that, in a high-risk “prediabetic” population the presence of reduced insulin sensitivity together with decreased insulin secretion jointly increase the diabetes development risk over time [36].

Similar were the results of studies in youth with early-onset diabetes [11,12,37,38,39,40,41,42] (Table 1). For example, the Treatment Options for type 2 Diabetes in Adolescents and Youth (TODAY) trial included 699 adolescents (aged 10–17 years) with T2D of <2 years duration in the United States. Participants were randomized to metformin alone, metformin plus rosiglitazone, or metformin plus lifestyle modification and were followed for 2–6 years. The study showed that the annual rate of β-cell decline in early-onset T2D ranged between 20% and 35% [12,43] and that treatment failed to prevent β-cell function deterioration, despite the absence of any insulin resistance increase. In another trial, the Restoring Insulin Secretion (RISE) study, interventions to improve or preserve β-cell function were compared across 91 adolescents (aged 10–19 years) and 132 adults (aged 20–65 years) with IGT or T2DM diagnosed within the previous 12 months [44]. Both groups were randomized either to three months of insulin glargine and nine months of metformin, or twelve months of metformin alone. A deterioration of β-cell function over 12 months was found despite treatment and a further deterioration after treatment withdrawal [45]. Similar were the results of another large epidemiological study designed to specify diabetes incidence and prevalence in the U.S., the so-called SEARCH for Diabetes in Youth study [46]. 

### 4.3. Studies Connecting Nutrient Levels with T2D Risk

Regarding micronutrients, several epidemiological studies have linked abnormal levels of vitamins, minerals, and trace elements with increased risk for T2D development (Table 2). For example, Pittas et al., in a systematic review and meta-analysis [47], suggested that calcium and vitamin D levels play a role in T2D development. More specifically, T2D prevalence was moderately but consistently associated with low vitamin D and calcium levels or decreased dairy intake, and, in addition, supplementation of subjects with these nutrients was associated with improved glucose metabolism. Similarly, a recent meta-analysis of epidemiological studies showed that an increase in dietary calcium intake to around 750 mg/day is inversely associated with T2DM risk [48], and other studies have associated an increased dairy product intake, and especially yogurt consumption, with decreased T2D risk [49,50]. Somewhat conflicting were the results of two different, but not directly comparable, studies which showed that higher circulating calcium levels are associated with an increased risk of T2DM [51,52]. 

Clinical trials on vitamin D supplementation in the general population have led to conflicting results regarding its effect on insulin secretion and islet cell function. It seems that in individuals with normal vitamin D levels, insulin secretion is not influenced by vitamin D supplementation as shown for example in a prospective double-blind randomized trial of obese Caucasian adolescents [53]. On the contrary, a higher 25[OH]D concentration at the baseline was independently associated with better β-cell function and lower glucose levels both cross-sectionally [54] and prospectively [55] in subjects at risk for T2D, suggesting that vitamin D supplementation could be a means of T2D prevention, as shown in the so-called Calcium and Vitamin D for Diabetes Mellitus (CaDDM) randomized controlled trial [56].

Another mineral that has been studied in relation to T2D risk is iron. Jiang et al. [57] followed 32,826 healthy women for 10 years in a prospective case-control study and showed that increased iron stores at the baseline (higher ferritin levels and a lower transferrin receptor to ferritin ratio) is an independent risk factor for T2D development [57], an observation consistent with the increased T2D prevalence found in cohorts of hemochromatosis and thalassemia patients [58]. Similarly, in a population-based study from Germany [59], it was shown that iron metabolism biomarkers (such as ferritin, transferrin, and soluble transferrin receptors, among others) are independently associated with impaired glucose metabolism and T2D. Nevertheless, specific data regarding whether an increased iron load affects β-cell function and insulin production or peripheral resistance and insulin action, or both, are scarce. 

Magnesium levels have also been implicated in T2D risk. For example, in a large prospective study, 85,060 women and 42,872 men with no history of diabetes, cardiovascular disease, or cancer at the baseline were followed. The authors identified a significant inverse relation between magnesium intake and T2D risk [60]. Similarly, a more recent study by Hata et al. [61] showed an inverse association between magnesium intake and T2D risk through improvement in inflammation and insulin resistance. Similarly to iron though, inadequate data exist to attribute to magnesium a protective role against T2D, either to improved insulin sensitivity, to ameliorated β-cell function and insulin production, or to both. 

Regarding selenium, which is another trace element, some studies have associated higher levels or an increased intake with higher T2D risk [62,63,64,65,66], even if the results are contradictory [67,68]. For example, a study by Wei et al. [63] evaluated the relationship between dietary selenium and T2D in middle-aged and elderly Chinese adults and showed a significant positive correlation between dietary selenium intake and T2D prevalence. Similar were the results of a cross-sectional analysis of 8,876 adults from the U.S. [62]. Furthermore, two large studies that examined the effect of selenium supplementation on T2D risk showed that selenium supplements failed to protect against T2D, and they can even increase the risk [64,65].

Finally, zinc has also been examined in relation to T2D risk and was found to have a mildly protective role. Sun et al. [69], for example, followed 82,297 women aged 33–60 years between 1980 and 2004 assessing their dietary intake of zinc, among other nutrients. It was found that higher zinc intake was associated with a slightly lower risk of T2D in women. More recently, Drake et al. [70] examined in a prospective study the effect of dietary and supplemental zinc intake on T2D risk in association with genetic polymorphisms in the *SLC30A8* gene, which codes for the zinc transporter-8 (ZnT8) protein. The authors found that zinc supplementation and a high zinc to iron intake ratio had a mildly protective role against T2D development, but these associations were weakened by obesity and specific *SLC30A8* genotypes.

### 4.4. Genetics, and Epigenetics Role in β-Cell Dysfunction

The importance of genetic predisposition to T2D is indisputable and has been confirmed by several epidemiological observations. Firstly, almost 40% of patients with T2D have at least one parent with the disease [71] while, in monozygotic twins, approximately 90% of the second twins will sooner or later develop T2D if the first suffers from the disease [72]. In addition, a first-degree relative of a patient with T2D has up to a 10-fold increased risk for diabetes development compared to the general population [73] and shows a reduced first- and second-phase insulin release before developing any insulin resistance [74]. 

**Figure 3:**
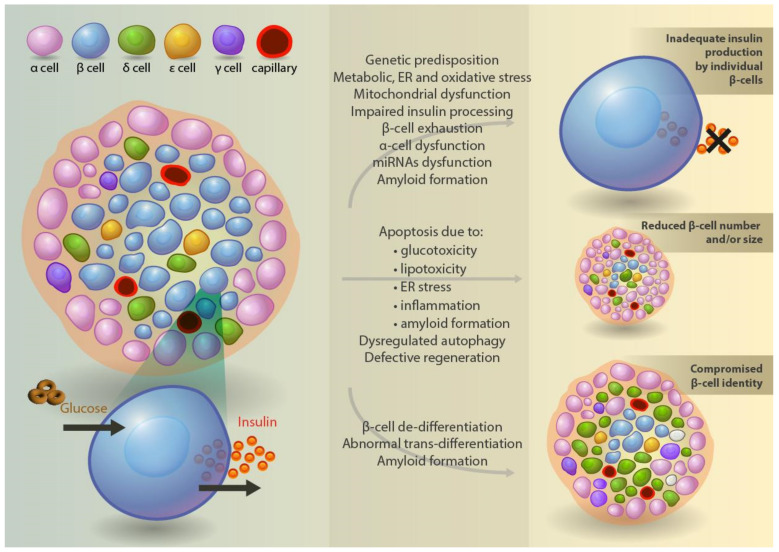
Factors that have been associated with inadequate insulin production by β-cells, with reduced β-cell number and/or size, and with compromised β-cell identity, ultimately leading to overt T2D.

Since T2D seems to result from a complex interplay between the genetic susceptibility of an individual and various environmental factors, a direct link between a specific genetic abnormality and a particular β-cell defect is difficult to establish [75]. Nevertheless, early studies have shown that genetic variants in at least three genes, namely the peroxisome proliferator-activated receptor-gamma gene (*PPARγ*) [76], the ATP-sensitive potassium channel Kir6.2 (*KCNJ11*) [77], and the gene encoding for transcription factor 7-like protein 2 (*TCF7L2*) [78] are associated with increased risks of developing T2D, especially in specific populations [79]. More recently, the introduction of large-scale genome-wide association studies (GWAS) has helped in identifying more than 700 distinct genetic loci that are strongly associated with T2D [80,81,82], a large proportion of which seems to influence insulin secretion (Figure 3). Gene variants that have been linked to increased T2D risk and defective insulin processing and secretion include variants close to *TCF7L2*, *HNF1A*, *HNF1B*, *SLC30A8*, *ADCY5*, and *MTNR1B* genes. Others, such as the ones close to *ARAP1*, *IGFBP2*, and *CCND2* genes have been also associated with increased T2D risk and are potentially linked to defective insulin synthesis [80,81,82]. 

Regarding youth-onset T2D, studies have begun to show a link between several established adult T2D genetic risk variants and increased risk for T2D in young subjects [83]. A recent study, for example, has associated the rs7903146 variant in the *TCF7L2* gene with an increased risk of IGT and T2D in obese adolescents by impairing β-cell function [84]. To better evaluate and make use of emerging data from GWAS and whole exome sequencing studies in youth with T2D, a scientific consortium has been developed, the so-called Progress in Diabetes Genetics in Youth (ProDiGY) [85].

Studies on genetic polymorphisms that associate nutrient abnormalities with higher risk for T2D have mainly focused on the vitamin D receptor (VDR) and on the ZnT8 protein. More specifically, several VDR polymorphisms, such as *FokI* and *BsmI*, have been associated both in children and in adults with defective insulin secretion and IGT, thus increasing the risk of overt T2D [86,87]. The results of studies in mice [88] and of a recent meta-analysis [89] confirmed the association between VDR polymorphisms and defective insulin secretion or susceptibility to T2D, thus further supporting the importance of vitamin D in β-cell function on one hand and the role of genetics in T2D development on the other. Regarding the *SLC30A8* gene that codes for the ZnT8 protein, a specific polymorphism has been identified in several studies and meta-analyses to be associated with an increased risk for T2D [90,91,92]. The ZnT8 protein colocalizes with insulin in the secretory granules of β-cells and is involved in the stabilization of zinc–insulin hexamers prior to their secretion. The importance of zinc and ZnT8 in insulin secretion is exemplified by the significantly decreased insulin secretion observed in mice with a β-cell specific deletion of the *SLC30A8* gene [93]. 

Further to the genetic predisposition to T2D, several studies have shown that both the intrauterine and early postnatal life environment may increase susceptibility of the individual to develop T2D later in life, possibly through epigenetic changes [94]. For example, a study in Pima nuclear families that compared siblings who were born before and after their mother was diagnosed with T2D, showed a 4-fold higher risk of diabetes development and a higher BMI for the offspring that was exposed to an intrauterine diabetic environment [95]. Similarly, fetal exposure to T1D mothers showed a 33% increased risk of IGT during adulthood compared to the offspring of T1D fathers [control group] [96]. These data imply that exposure of the fetus to maternal diabetes increases the risk of abnormal glucose management later in life, regardless of the possible effect of genes associated with early-onset T2D. These findings were corroborated by more recent data from the TODAY study [97]. Furthermore, it seems that β-cell function, rather than insulin sensitivity, is mostly affected in the offspring of women with early-onset T2D [98,99], and that such decreased function is present even in individuals with normal glucose tolerance (NGT). Besides gestational diabetes, prenatal nutrient insufficiency leading to low birth weight (small for gestational age -SGA-) has been associated with increased susceptibility to T2D [100], even if the molecular pathomechanisms involved are still under investigation [101] and both insulin resistance [102] and β-cell dysfunction have been shown to be implicated [103,104]. Especially regarding the latter, studies have demonstrated that obese children born SGA demonstrate impaired β-cell function compared to normal birth weight or obese counterparts [103], as well as a reduction in pancreatic endocrine cell mass after birth [104]. 

### 4.5. Defective Insulin Secretion from β-Cells before, at the Time of, and after T2D Diagnosis

In patients with T2D, the defective secretion of insulin by the pancreatic islets relative to the prevailing glucose concentrations could be either due to the decreased secretory rate of the individual β-cells, due to the reduced total β-cell number and/or size, or due to a combination of these two factors (Figure 3) [17,18,19]. In vitro studies examining the relative contribution of each of these two factors have several intrinsic difficulties. Nevertheless, several experimental studies have been carried out starting in the early 1980s which have mostly shown a profound decline in β-cell insulin production and secretion. In an early paper by Fernandez-Alvarez et al., for example [105], it was shown that insulin release was decreased in diabetic compared to non-diabetic islet preparations. In another study involving perifusion assays, it was shown that insulin release by diabetic β-cells is decreased and is triggered by a higher glucose threshold [106]. Using different macronutrients (glucose and the amino-acid arginine) and glibenclamide as stimuli, Guerra et al. [107] showed the inability of T2D β-cells to adjust insulin secretion to increasing glucose levels. Furthermore, since insulin secretion from the diabetic islets was better preserved after glibenclamide and arginine stimulation, the authors suggested that a stimulus–secretion uncoupling is a more plausible explanation than decreased insulin content or defective insulin granule exocytosis.

Clinical studies on prediabetes and T2D in youth have shown a significant β-cell dysfunction before or at the time of T2D diagnosis that rapidly deteriorates in combination with an increasing insulin resistance [108,109,110,111,112]. In addition, one must keep in mind that puberty is characterized by a transient reduction in insulin sensitivity by almost 30–50% in lean, healthy children [113]. The increased insulin secretion needed to compensate for this physiological change can be difficult to meet in adolescents with restricted β-cell function due to genetic, epigenetic, or environmental factors, and therefore puberty can be considered as a high-risk period for T2D development in such subjects. A study by Weiss et al. [114] showed that obese youth with IGT have a defective first-phase insulin secretion, while a decreased second-phase secretion is specific for T2D. On the contrary, a recent cross-sectional analysis of RISE Study data showed higher insulin secretion rates and β-cell secretion more responsive to glucose in youth with IGT or T2D relative to adults even after adjusting for insulin resistance differences [115]. Abnormalities in the insulin secretion pattern, such as a diminished or absent first phase insulin release after a glucose load [74,116,117] and defects in its pulsatility cycles [118,119] have been also reported in studies with adult T2D patients. 

As obesity and metabolic derangements deteriorate and prediabetes progresses to overt T2D, defective β-cell insulin production becomes more and more pronounced. It has been shown, for example, that the expression of genes involved in oxidative stress management decrease in T2D β-cells [120], making glucotoxicity, lipotoxicity, oxidative stress, and ER stress key players in the progressive β-cell impairment of T2D [121,122,123,124]. In addition, the function of ion channels has been shown to deteriorate due to T2D stress leading to perturbations in their activity, expression, or localization [125]. Furthermore, several lines of evidence in adults support that a defective incretin effect, most probably due to an impaired β-cell response especially to GIP, appears early in the prediabetic state, deteriorates as glucose intolerance develops, and becomes nearly total in overt T2D [126,127]. Regarding youth-onset T2D, Arslanian et al. [128] showed that glucose sensitivity in obese youth decreases across the spectrum of glucose tolerance parallel to an incretin effect impairment without reduction in GLP-1 or GIP. Data on which nutrients could influence the incretin effect in T2D patients are scarce. In a recent study [129], Kahleova et al. compared two energy- and macronutrient-matched meals, a standard meat and a vegan meal, in patients with T2D. The authors showed that incretin and insulin secretion were higher after the vegan meal, suggesting that plant-based meals could be used to improve glucose handling in patients with T2D. 

### 4.6. Defective Insulin Production, Processing, and Secretion at the Cellular Level

Several studies have shown the importance of various micronutrients in insulin production and secretion by β-cells. Normally, insulin is stored in the secretory granules together with zinc, one of the most abundant trace elements in the human body. Zinc plays an essential role in the process of insulin secretion, with two zinc ions forming the center of each insulin hexameric structure that is secreted into the portal circulation in response to a secretory stimulus [130,131]. Further to this role, zinc may be important for normal β-cell insulin production since it has been shown that depletion of intracellular zinc in experimental conditions is linked with a more than 50% decrease in insulin secretion [132]. On the contrary, zinc concentrations in the extracellular space around β-cells have been shown to act in an autocrine negative feedback loop to decrease insulin secretion [133], and, similarly, zinc over-administration decreases insulin gene expression and diminishes β-cell viability [132,134]. Observational and experimental studies have shown that moderate zinc supplementation is associated with a decreased T2D risk in women [69], with improved glucose regulation in patients with T2D [135], and in mice [136]. It must be mentioned, though, that these effects may not only be mediated through zinc’s beneficial role on insulin storage and secretion, but also through improved glucose uptake by adipocytes [137]. 

Another important dietary mineral involved in insulin production and action is chromium [138]. Indeed, early studies have shown that dietary chromium increases insulin sensitivity and has been therefore named as a glucose tolerance factor [139]. More recently, a systematic review of randomized controlled trials showed that chromium supplementation has no effect on healthy people but significantly improves glucose metabolism in people with T2D [140]. Nevertheless, it is not well established whether these chromium effects are only mediated through an increase in insulin sensitization or whether an improved insulin production by β-cells is also involved. A study with an experimental diabetic rat model showed that oral chromium picolinate administration attenuated hyperglycemia-induced oxidative stress and probably enhanced insulin production by β-cells [141]. 

In addition to zinc and chromium, vitamin D has been shown in several studies to play an important role in normal insulin secretion. For example, an early study by Katowaki et al. [142] showed that in the perfused rat pancreas, vitamin D acts directly in mediating insulin secretion and not through changes in calcium concentration. More recent studies have shown that vitamin D mediates its effects through its nuclear receptor VDR and that mice lacking VDRs demonstrate decreased insulin mRNA content and subsequent defective insulin biosynthesis [88], even if not all relevant studies corroborate these findings [143]. In a recent study by Bornstedt et al. [144], the glucose-stimulated secretion of insulin by INS1E β-cells was shown to be correlated with the type of vitamin D molecule used, since treatment with 1,25(OH)2D led to increased insulin production while 25(OH)D did not. Even though vitamin D’s role in β-cell insulin production is indisputable, clinical trials on vitamin D supplementation in the general population have led to conflicting results regarding its effect on insulin secretion and islet cell function. It seems that in individuals with normal vitamin D levels, insulin secretion is not influenced by vitamin D supplementation [53]. 

Since iron has been shown to play an essential role in glucose–insulin coupling as well as in all stages of insulin production and secretion [145], it is no wonder that disturbances in its concentration have been linked to defective insulin production and T2D. For example, several studies have shown an increased risk for T2D not only due to increased insulin resistance but also due to β-cell dysfunction in patients with hereditary hemochromatosis and diseases associated with iron overload due to frequent transfusions such as thalassemia major [58,146,147,148]. Conversely, decreasing iron overload in these patients leads to better glycemic control due to improved β-cell function and increased insulin production [149,150]. On the other hand, a very recent study by Qin et al. [151] compared β-cell function and insulin sensitivity relative to iron metabolism parameters, between patients newly diagnosed with T2D and healthy lean control subjects. The study showed that in patients with T2D, serum ferritin was independently associated with impaired β-cell function, while insulin sensitivity was not affected by iron levels. In addition, transferrin levels were shown to play a protective role for β-cell function in male patients. In a before-and-after study, the administration of a single oral dose of iron in healthy male volunteers was associated with an acute iron-induced impairment in β-cell function and insulin secretory capacity [152]. 

One must remember though, that not only excessive, but also deficient iron can lead to derangements in β-cell function through various mechanisms. In a study including patients with T1D, those with an iron deficiency had increased glycated hemoglobin compared to those with normal iron levels, suggesting that low iron levels could negatively affect the few β-cells remaining active in T1D [153]. Furthermore, in an experimental model of mice that lack a regulator of cellular iron homeostasis, namely iron-regulatory protein 2, leading to functional iron deficiency in their β-cells, Santos et al. [154] showed a defective processing of proinsulin to mature insulin, leading to reduced insulin levels and glucose intolerance. When treated with iron, these mice restored their glucose tolerance through increased insulin production. The authors identified a newly described mechanism interconnecting intracellular low iron levels and defective insulin processing that could be of importance in abnormal glucose metabolism [155].

Further to the influence of nutrients on insulin secretion, various changes in insulin processing have been described in patients with T2D. In healthy individuals, insulin is initially produced as proinsulin that is processed and finally cleaved to mature insulin and c-peptide. Up to 10–15% of the total insulin secreted by β-cells is in the form of proinsulin. On the contrary, it has been shown by Kahn et al. [156] that the proportion of proinsulin secreted in individuals with T2D is considerably increased in the basal state, reaching more than 40% of the total amount. This difference in proinsulin secretion persists even after matching for obesity degree, implying that it probably corresponds to a dysfunction of β-cells rather than an adaptive response to the increased demands of obesity-associated insulin resistance [157]. In youth with T2D, an early study by Weiss et al. [114] demonstrated that proinsulin to insulin ratios during first- and second-phase secretion were similar for subjects with NGT and IGT but were markedly increased in T2D. The authors concluded that a defect in proinsulin processing is characteristic for young patients with T2D. In another more recent study, it was shown that children and adolescents with impaired glucose regulation had significantly elevated proinsulin levels, both during fasting and after glucose stimulation, pointing to a β-cell dysfunction [158]. Furthermore, proinsulin, and proinsulin to insulin ratio, have been shown to be independent predictors of early glycemic control loss in a very recent study based on data from the TODAY study [159].

### 4.7. β-Cell Mass in Type 2 Diabetes

When assessing and describing the quantity of β-cells in the islets of non-diabetic or T2D subjects, either the β-cell mass can be used when the weight of the sample is available, or its volume, presuming that the islets have a spherical shape. Alternatively, if a special staining method is used, the area of the pancreatic islet that is insulin-positive is used as an indicator of the amount of β-cells. The quantity of β-cells has been found to be decreased in pancreatic samples of patients with T2D, no matter which of the above methods is used and this decrease has been shown to be progressive as the disease evolved [160,161,162,163,164]. Several mechanisms have been implicated in this β-cell mass reduction such as amyloid deposition and increased fibrosis [160], increased α-cell proportion in the islets [162], as well as accentuated β-cell apoptosis [163]. Studies examining β-cell mass at the time of appearance or during the progression specifically of early-onset T2D in youth have not been published [165]. 

Regarding the effect of different nutrients on the pancreatic β-cell mass, data are scarce. For example, vitamin A has been shown to be involved in the development of fetal pancreatic islets, and its deficiency during the intrauterine life leads to glucose intolerance in adult rats [166]. Another study showed that vitamin A is essential in maintaining adequate β-cell mass in adult rats as well, since experimental animals fed diets poor in vitamin A show islet remodeling and increased β-cell apoptosis, leading to decreased β-cell mass and smaller islet size [167].

Despite the wealth of data suggesting that β-cell mass decrease has a key role in T2D pathogenesis (Figure 3), some studies have shown an overlap in the β-cell mass measured between T2D patients and controls, especially in lean diabetic subjects [168]. In addition, a recent study that assessed β-cell loss by insulin immunostaining under light and electron microscopy questions the real extent of β-cell loss, since normal degranulated cells that were identified by electron microscopy were missed by light microscopy, and thus were considered to be abnormal [169]. Similarly, for the β-cells that are identified by insulin staining, only those β-cells with adequate insulin content will be identified and counted, leading to a possible underestimation of β-cell mass. On the contrary, since β-cell hypertrophy has been identified in patients with T2D, the loss of β-cell number could in some cases be more profound than what has been reported [170].

### 4.8. Mechanisms of β-Cell Death and Regeneration

The decreased β-cell mass that characterizes T2D can be attributed to an imbalance between the higher death and lower regeneration rate (Figure 3) [163,171,172]. The main mechanism through which β-cell death occurs is apoptosis, a recognized mode of “programmed” cell death. β-cell apoptosis can be initiated by several factors, such as chronic low-grade inflammation that is typical for T2D, oxidative stress due to high levels of glucose and free fatty acids (FFAs) in the blood—the so-called lipotoxicity and glucotoxicity, and ER stress due to the accumulation of misfolded proteins. In addition, genetic mutations, such as mutations in the genes encoding for the transcription factor HNF1A and the ATP-sensitive K^+^ channel, have been linked to impaired β-cell function and apoptotic initiation [173,174].

One of the largest studies on β-cell mass and death in T2D, [163], showed that apoptosis was markedly higher in both obese and lean patients with T2D compared to BMI-matched non-diabetic controls and led to a β-cell mass reduction of up to 40–50%. In another study by Hanley et al. [168], β-cell apoptosis was 3-fold higher in the pancreatic tissue of obese patients with T2D compared to matched non-diabetic controls. Furthermore, a report on islets isolated from T2D subjects showed increased apoptosis with enhanced caspase-3 activity, which was partially reversed after a 24 h incubation of the T2D islets with metformin [175]. Interestingly, a recent study in mice demonstrated that vitamin D treated β-cells show decreased apoptosis leading to enhanced insulin secretion [176]. In addition, it seems that in patients with T2D there is a close connection between elevated FFAs, oxidative stress and increased ROS production, impaired ER and mitochondrial calcium homeostasis, and β-cell functional impairment leading ultimately to their apoptosis [177].

Despite its importance, apoptosis is not the only way of programmed β-cell death in patients with T2D [178,179]. Autophagy is a programmed cell process which, under normal conditions, is crucial for the physiological degradation of senescent or damaged organelles and proteins [180]. However, in T2D hyperglycemic conditions, autophagy has been shown to become dysregulated leading to accelerated β-cell death in a non-apoptotic manner [181,182,183]. In a study that examined human pancreatic samples under electron microscopy [184], dead β-cells with morphological evidence of autophagy-associated cell death were more in the diabetic vs. the non-diabetic samples. In selected cases of youth with phenotypic T2D, the occurrence of pancreatic autoantibodies leading to β-cell destruction and death has been also described [185]. 

Iron dysregulation has been linked with accelerated β-cell death in patients with T2D through several mechanisms but mainly through its important effect on reactive oxygen species (ROS) formation and accentuated oxidative stress [186]. In addition, iron overload can lead to β-cell dysfunction and death through so-called ferroptosis. Ferroptosis is a non-apoptotic form of regulated cell death, caused by glutathione depletion which leads to inhibition of the enzyme glutathione peroxidase-4 and subsequent lipid ROS accumulation [187]. β-cell defense mechanisms against oxidative stress can be further compromised in case of an iron overload through its effect on ROS detoxification enzymes that involve other trace elements as well, such as mitochondrial manganese uptake and manganese-dependent superoxide dismutase (SOD) activity [188].

Increased cell death is not the only culprit responsible for decreased islet cell mass in T2D since defects in β-cell regeneration may also be of significance. The possible mechanisms through which the human pancreas can “regenerate” insulin producing cells, albeit at a very slow rate [189,190], is by proliferation of existing β-cells, by neogenesis from precursor cells, and by trans-differentiation of other mature cells [191,192,193]. Relevant studies with pancreatic tissues from T2D patients have been quite difficult and have presented conflicting results [168,194]. 

### 4.9. Trans- and De-Differentiation of β-Cells

Trans-differentiation refers to the process through which various mature pancreatic cells become insulin producing cells in an attempt to increase the β-cell mass of a patient with T2D [193,195,196]. Human pancreatic tissue sample studies have identified cells with both glucagon and insulin, even in very small numbers, suggesting possible interconversion between different cell types [197,198]. In addition, evidence of islet plasticity in humans comes from studies showing that at 13–25 gestational weeks, up to one tenth of the pancreatic endocrine cells co-express glucagon and insulin [199]. At this early stage of pancreas development, vitamin D seems to play an important role in islet cell proliferation and differentiation since vitamin D receptors have been shown to be expressed in human fetal pancreatic progenitor cells and to be responsive to calcitriol [200]. In addition, it has been shown that transretinoic acid, which is the predominant biologically active form of vitamin A, plays an essential role in the development of progenitor cells that will differentiate into β-cells, by acting on the retinoic acid receptor [201]. 

Studies on subjects with abnormal glucose levels have shown an increased number of bi-hormonal cells (i.e., cells containing both glucagon and insulin), especially in prediabetic or early diabetic states [194,197,198]. Other sophisticated in vivo and in vitro studies have shown that adult cells either of the exocrine or the endocrine pancreas, such as acinar, ductal, and α-, γ-, and δ-cells show an unexpected plasticity and can, after appropriate manipulation, be trans-differentiated into insulin producing β-cells [195,196,202,203]. It is still uncertain to which extent this trans-differentiation plays a protective role during the early stages of T2D and if, gradually, the failing of this mechanism aggravates pancreatic insufficiency leading to overt T2D. 

Opposite to the above-described trans-differentiation, de-differentiation refers to the gradual loss of β-cell molecular and functional identity which leads to gradual insulin production insufficiency and increased cell death rates (Figure 3) [204,205,206]. Of interest, de-differentiation seems to be, at least in part, mediated by glucotoxicity, and lowering glucose levels with appropriate treatment has been associated with some β-cell function restoration [207]. Other stressful conditions, such as the lipotoxicity and inflammation that characterize T2D, have been shown to further aggravate β-cell de-differentiation [205,208]. Studies in cell cultures, experimental animals, and in humans have indicated that β-cell de-differentiation could be an important mechanism involved in decreased insulin containing and producing pancreatic cells in T2D [209,210,211]. Furthermore, aging has been shown to accentuate β-cell de-differentiation irrespective of other factors. For example, Song et al. [212] identified a 2-fold increase in the proportion of de-differentiated cells in elderly and middle-aged nondiabetic subjects compared to younger ones, which was mainly attributed to a defective unfolded protein response.

Regarding micronutrient levels, calcium homeostasis seems to play an important role not only in β-cell function and insulin production, but also in their viability, replication, and differentiation. Conversely, the chronic stimulation of intracellular calcium signaling pathways leads to ER stress, increased inflammation, and β-cell de-differentiation [213]. That could partly explain the observed increased risk for T2D in patients with high circulating calcium levels [51,52]. 

### 4.10. Role of Islet Inflammation

Both macrophages and cytokines under normal conditions have been shown to support β-cell proliferation and function [214,215,216]. On the contrary, patients with obesity and T2D present with chronic inflammation in their pancreatic islets, as evidenced by the infiltration of a high number of inflammatory cells and local release of cytokines and chemokines (Figure 3) [217,218,219,220,221]. Not only the number but also the phenotype of macrophages seems to favor an inflammatory milieu, since within the islets of at least two different T2D mouse models, macrophages were shown to shift to an inflammatory M1-like phenotype, the so-called M1 polarization [222,223]. 

Studies regarding the role of inflammation in early-onset T2D in youth are scarce and have mostly examined inflammatory markers that are obesity-related and possibly promote insulin resistance. Such pro-inflammatory cytokines include TNF-α, IL-6, IL-1β, interferon-gamma (IFNγ), pigment epithelium-derived factor (PEDF), and fetuin A while decreased adiponectin and omentin have also been implicated in diminished insulin sensitivity [224,225,226]. Many fewer studies have shown an association between specific cytokines and impaired β-cell function in youth T2D including increased TNF-α [227], increased fetuin A [228], and a decrease in the concentration of the anti-inflammatory cytokine fibroblast growth factor-21 (FGF-21) [228]. Despite the lack of concrete evidence, the pro-inflammatory milieu that characterizes central obesity could be blamed, at least in part, for the rapid progression of T2D in adolescents and young adults compared to later-onset T2D. Indeed, obesity is a more common feature of early-onset T2D with studies reporting that >80% of youth with T2D are obese at presentation compared with 50% of adults with the disease [229,230].

Regarding the role of micronutrients in T2D inflammation, several studies have implicated various vitamins, minerals, and trace elements. For example, it has been shown that vitamin D has a protective role against inflammation-induced β-cell dysfunction, possibly through changes in cytokine concentrations in the islets of pancreas [231,232], among other mechanisms [233]. In addition, a study in an animal model of experimental diabetes showed that cobalt administration led to decreased oxidative stress, restored nitric oxide synthase equilibrium, and ultimately improved cellular function [234]. 

Another mineral with an important antioxidant role in patients with T2D is zinc. Zinc has been shown to modulate metallothionein expression and glutathione metabolism, to act as a superoxide dismutase cofactor, to compete with iron and copper in the β-cell membrane, and to inhibit enzymes involved in the inflammatory process [235]. Indeed, studies with zinc supplementation in overweight T2DM patients have shown an improvement in disease markers through enhancement of their total antioxidant capacity [236,237]. In addition, a study with rats made diabetic by injecting a single streptozotocin dose showed that the combined administration of zinc, selenium, and vitamin E had the best anti-oxidative result and improved glycemic control more than the antidiabetic drug glibenclamide [238].

Magnesium has also been shown to act as an antioxidant in both experimental and clinical conditions. For example, in a study with diabetic mice, magnesium administration for 6 weeks improved mitochondrial function and reduced mitochondrial ROS production and calcium overload, leading thus to diminished oxidative stress [239]. 

A similar action has been attributed to selenium in experimental conditions, even if epidemiological studies have linked selenium supplementation with increased T2D risk. More specifically, a study investigating selenium administration to non-obese diabetic mice for 3 weeks was associated with improved glycemic control, decreased oxidative stress, and diminished apoptotic death [240]. Since selenium has been shown to be essential for glutathione peroxidase enzyme activity, an important cellular defense mechanism against ROS, it seems plausible that this trace element might play an important role against the oxidative burden of T2D, when present at the appropriate concentrations in the body [67,241].

### 4.11. Role of α- and Other Islet Cell Dysfunction in Diabetes Pathogenesis

Several lines of evidence suggest that glucose homeostasis is tightly controlled by a delicate balance between insulin production from β-cells and glucagon production from α-cells and that this balance loss could be a key player in T2D pathogenesis (Figure 3) [242,243]. For example, an elegant experiment performed by Lee et al. showed that, in mice with β-cell destruction by high dose streptozocin, only those with intact liver glucagon receptors developed fatal diabetic ketoacidosis. Glucagon receptor-null mice remained clinically normal after similar β-cell destruction without increased blood levels, impaired glucose tolerance, or hepatic glycogen depletion [244].

In normal conditions, glucagon is known to increase insulin secretion in the postprandial phase but not when it is secreted in response to hypoglycemia. Reciprocally, under normal conditions, insulin has been shown to regulate glucagon secretion [245]. Studies in adult patients with T2D have shown a glucagon overproduction from α cells both while fasting and after a carbohydrate-rich meal, in response to an intravenous glucose load, and to arginine administration, thus suggesting an impaired α-cell regulation that could accentuate hyperglycemia [246,247]. Studies in youth are scarce with conflicting results regarding the possible role of α-cell dysregulation in β-cell dysfunction and youth T2D pathogenesis [248,249]. 

Similarly scarce are data on the effects of nutrient dysregulation on α-cell dysfunction and T2D pathogenesis. A recent study by Trasino et al. [167] showed that feeding adult rats with a vitamin A-deficient diet not only leads to increased β-cell apoptosis and decreased β-cell mass, but also increases α-cells and causes hyperglucagonemia, thus disturbing the cellular balance within the islets and further aggravating hyperglycemia.

### 4.12. Role of Islet Amyloid Polypeptide

Islet amyloid polypeptide (IAPP, also known as amylin), is a peptide protein that is synthesized and stored in β-cells in insulin secretory granules and is secreted together with insulin, in one-tenth to one-hundredth of the amounts of insulin [250]. Early studies had shown that the presence of amyloid plays a role in β-cell mass reduction in patients with T2D [251,252]. It gradually became evident that these amyloid deposits are mainly formed by IAPP aggregation [253]. More recent studies have shown that not all forms of IAAP aggregates trigger β-cell damage and death, but it is mainly IAAP toxic oligomers which penetrate the β-cell membrane, cause its destabilization, and contribute to β-cell damage and apoptotic death [254]. 

Amylin has been shown to exert an autocrine action, increasing the β-cell proliferation rate at low, and decreasing it at high, glucose levels [255]. Furthermore, high concentrations of IAPP within the islets have been linked to decreased glucose uptake by β-cells and subnormal insulin secretion [256,257], suggesting that it could be directly involved not only in increased β-cell death but also in decreased insulin production by the surviving β-cells. In addition, IAAP aggregates cause ER stress and mitochondrial damage [258]. Interestingly, a specific missense mutation of the amylin gene (the S20G mutation, i.e., change of serine to glycine at position 20) has been shown to be, at least partially, associated with diabetes pathogenesis in Japanese patients with T2D [259].

Regarding micronutrients, iron has been implicated in IAPP formation and deposition [260]. In addition, iron in the form of heme has been shown to form a complex with IAPP which leads to hydrogen peroxide formation and β-cell death through oxidative stress [261,262]. On the contrary, both zinc and vitamin C have been shown to protect β-cells from amylin formation making their deficiency a possible mechanism implicated in T2D pathogenesis [259]. Indeed, studies on the role of vitamin C (ascorbic acid) in patients with T2D have been carried out or are ongoing [263], and some [264,265] but not all [266] have demonstrated that vitamin C supplementation can improve fasting blood glucose levels and glycosylated hemoglobin.

### 4.13. Role of miRNAs Dysfunction

MicroRNAs (miRNAs) are endogenously produced, short non-coding RNA molecules that play an important role in regulating gene expression by suppressing mRNA translation. During the last two decades, miRNAs have been identified as systemically controlling β-cell development, identity preservation, survival, and function, and to play an important role in its malfunction and apoptotic death in T2D, as well as to affect insulin synthesis and secretion both in human islets and in animal models (Figure 3) [267,268,269,270,271,272]. In mice, for example, miR-375 is highly expressed in healthy β-cells regulating the expression of several genes that are implicated in normal insulin secretion, such as Glut2, which is involved in glucose uptake, and Pdx-1, which plays a key role in β-cell function [271]. At the same time though, miR-375 has been found to be dysregulated both in experimental [273,274] and real-life conditions related to T2D [275]. In addition, other malfunctioning miRNAs have been found to play a role in β-cell failure and T2D pathogenesis, such as miR-7, miR-9, miR-29, and miR-30d, among several others [272,276]. No studies have been published so far regarding the possible association between micronutrients and miRNA levels in patients with T2D, but a recent study by Ferrero et al. [277] showed a link between habitual diet nutrients and circulating miRNA profiles in healthy subjects. More research is, therefore, warranted.

## 5. Conclusions

This extensive narrative review summarized the pathomechanisms that have been shown to be involved in adult- and early-onset T2D, especially in conjunction with the intake and body levels of specific vitamins and minerals. Some of these mechanisms have been studied extensively in adults while, for others, new data are emerging from ongoing studies. Data on specific nutrients are scarce regarding their effects on β-cell function, especially in youth-onset T2D. The severe β-cell dysfunction even at the time of diagnosis in younger patients with T2D, together with their rapid function decline, make youth-onset T2D an aggressive disease with special characteristics and, possibly, with more factors involved in its pathogenesis. Further research is needed to elucidate such factors. For example, data gathered from the RISE and TODAY studies could be further analyzed regarding additional differences related to puberty stage, race and ethnicity, adipose tissue distribution and body composition, nutrient intake and so on. In addition, important information provided from prospective studies on cohorts of youth with T2D will help better understand youth-onset T2D natural history and will determine risk and protective factors for β-cell deterioration [278]. Since micronutrient intake can be easily manipulated, nutrients in particular could be an excellent target for T2D prevention and management, if only more reliable data became available [279]. 

## Figures and Tables

**Table 1:** Various pathomechanisms that have been implicated in β-cell failure in youth-onset vs. adult-onset T2D. For relevant references, please refer to the main text.

	Adult-Onset T2D	Additional Factors Related to Youth-Onset T2D
Genetic traits	*PPARγ*, *KCNJ11*, *TCF7L2* variants from earlier studies, >700 loci from GWAS studies	No studies linking genetic traits with T2D specifically in youth populations
Early life and epigenetics	Intrauterine diabetic environment (mother’s gestational diabetes)	Corroborated by data from the TODAY study on youth populations
	Prenatal nutrient insufficiency leading to SGA	
Decreased secretory rate by the individual β-cells	Present already at the time of diagnosis (possibly due to several mechanisms)	Identified in youth studies together with a much more rapid deterioration in β-cell function
	Defective first and second phase insulin secretion	
Impaired insulin processing	Increased proinsulin to insulin ratio	Identified in youth studies too
Reduced β-cell mass	Both islet volume β-cell density and total β-cell mass decreased at the beginning and gradually progressed	No studies specifically in youth populations
Increased β-cell death and lower regeneration rate	Increased apoptosis due to several mechanisms (glucotoxicity, lipotoxicity, ER stress, oxidative stress etc.)	No studies specifically in youth populations
	Increase autophagy	
	Defective regeneration	
Trans- and de-differentiation of β-cells	Defective trans-differentiation may play a role. More robust data on increased de-differentiation of β-cells	May be important due to high rates of obesity and increased inflammation in youth with T2D, but no studies have been published
Pancreatic islet inflammation	Macrophages and cytokines important under normal conditions for β-cell function, but participate in their malfunction in T2D	Scarce studies mainly about obesity-related inflammatory markers. Must be important in the obesity-driven inflammatory milieu of youth-onset T2D
Role of α- and other islet cell dysfunction	Increased α-cell function and hyperglucagonemia has been implicated in T2D pathogenesis	Scarce studies with conflicting results regarding α-cell function
Islet amyloid polypeptide accumulation	Mainly IAAP toxic oligomers have been incriminated in defective insulin production	No relevant studies in populations with youth-onset T2D
Role of miRNAs dysfunction	Involved in β-cell development, identity preservation, survival, and function but also in their malfunction and apoptotic death in T2D (e.g., miR-375)	No relevant studies in populations with youth-onset T2D

**Table 2:** Specific vitamins and minerals/trace element derangements that have been linked with a protective or harmful effect on β-cell function in type 2 diabetes. For relevant references, please refer to the main text.

	Factors Identified to Have a Protective Role	Factors Identified to Have a Harmful Effect	Comments
Vitamin D	Vitamin D supplementation associated with improved glucose metabolism	Low vitamin D levels as well as specific VDR polymorphisms associated with increased T2D risk	Higher levels and supplementation seem to play a protective role only in subjects at risk for T2D, possibly through decreased inflammation
Vitamin A		Experimental animals fed vitamin A-poor diets showed increased β-cell apoptosis, decreased β-cell mass, and increased α-cells	Essential in maintaining adequate β-cell differentiation and mass in experimental conditions
Vitamin C	Some studies have linked vitamin C supplementation with decreased fasting blood glucose levels and glycosylated hemoglobin		May have a protective role against amylin formation
Calcium	Calcium supplementation associated with improved glucose metabolism	Low calcium levels or decreased dairy product intake	Conflicting results from some studies showing that higher calcium levels increase T2D risk
Iron		Higher levels are independently associated with impaired glucose metabolism and T2D. Possibly lower levels too	Not clear if β-cell function is mostly affected, peripheral insulin sensitivity, or both
Magnesium	Intake linked with decreased T2D risk		Possibly acts as an antioxidant. Not clear if β-cell function is improved, peripheral insulin sensitivity, or both
Selenium	Some studies have linked selenium intake with lower T2D risk	Others have associated higher levels or increased intake with higher T2D risk	Role not clear, possibly acts as an antioxidant when in the right concentration
Zinc	Zinc intake seems to have a mildly protective role against T2D and better glycemic control in women, patients with T2D, and in experimental animals	Specific *SLC30A8* gene polymorphisms (codes for ZnT8 protein) have been linked with higher T2D risk	Its action may be influenced by several factors such as obesity and specific genetic traits. Important role in insulin hexamers. Possibly acts through decreased inflammation
Chromium	Chromium supplementation improves glucose metabolism in people with T2D		Not clear if β-cell function is improved, peripheral insulin sensitivity, or both

## Data Availability

Not applicable.
